# University Teachers During the First Lockdown Due to SARS-CoV-2 in Italy: Stress, Issues and Perceptions of Misconduct

**DOI:** 10.1007/s11948-022-00362-9

**Published:** 2022-02-15

**Authors:** Oronzo Parlangeli, Paola Palmitesta, Margherita Bracci, Enrica Marchigiani, Ileana Di Pomponio, Stefano Guidi

**Affiliations:** Department of Social, Political and Cognitive Sciences, University of Siena, Via Roma 56, 53100 Siena, Italy

**Keywords:** Academic, University teachers, Faculty members, Pandemic, Stress, Misconduct, Gender

## Abstract

With the spread of the pandemic and the introduction of measures aimed at its containment, it is necessary to understand in specific national contexts how home quarantine has affected the psychophysical well-being of academics, and their ability to maintain integrity. To this end we constructed an online questionnaire to investigate the levels of stress, well-being, and work-life balance in relationship with living and working conditions. Moreover, the questionnaire was designed to obtain information about the perceived occurrence, increase or decrease of misconduct in research (e.g., research misconduct by colleagues) and professional relationships (e.g., misbehaviors between colleagues, from students and toward students). The questionnaire was administered online by contacting faculty at three universities in Tuscany, Italy, asking them to relate their experience during the first lockdown (March–May 2020). Faculty members were invited to complete the questionnaire by their institutional e-mail account. The final sample consisted of 581 respondents. The results showed that inadequacies of the equipment, and particularly poor internet connection, were significantly correlated with main issues reported, such as relationships with students and research activities. Female teachers primarily suffered from stressful conditions, lacked well-being, and experienced work-life imbalance. Stress levels were related to perceptions of the frequency of misconduct and of an increase in their frequency during the period of home quarantine. Female professors, when compared to their male counterparts, perceived misconduct from students as increased and more frequent in the period of quarantine. Results point to a gender issue that is likely to arise from conditions of domestic activities imbalance and that increases stress and misconduct perception.

**Supplementary Information:**

The online version contains supplementary material available at 10.1007/s11948-022-00362-9.

## Introduction

In late 2019 and early 2020 (Wu et al., 2020), the spread of the new coronavirus SARS-CoV-2 raised concerns that many sectors—from health care to transportation, from industry to technological infrastructure—were not adequately prepared to deal with the outbreak. As the pandemic evolved and rapidly took on dramatic proportions, several countries were faced with the need to adopt containment measures. First among all Western countries, starting from the 9th of March 2020 (Lazzerini & Puoto, 2020), Italy established a first, strict, lockdown period that was then extended until May 2020.

The condition of isolation can lead to various negative consequences for physical and psychological health, with the manifestation of various symptoms that very commonly result in increased levels of stress and anxiety (Cao et al., 2020; Okruszek et al., 2020; Wang et al., 2020). These problematic symptoms can also progress to conditions of mental illness (Moccia et al., 2020) which can continue for much longer than the duration of their causing event (Fiorillo & Gorwood, 2020; Galea et al., 2020).

The educational system was among the sectors most affected by the condition of isolation. Institutions of higher education have been forced to face the spread of the pandemic by adopting various measures, from the interruption of research activities and in-person classes to the push for their employees towards distance working (Quattrone et al., 2020). This has resulted in changes in activities and social relationships which, regards to students, have been and still are evident and widely studied (see, for example, Gualano et al., 2020; Sun et al., 2021), thus showing, for instance, that symptoms’ severity is associated with Covid-19 social stigma and that females are more likely to develop anxious symptoms (Sun et al., 2021) and distress (Maugeri et al., 2020).

If university students, in Italy and around the world, have been taken into consideration by research, the same attention has not been devoted to faculty members. Actually, studies conducted prior to the pandemic suggest that faculty in an academic environment may inherently be subject to conditions that induce high levels of stress (Catano et al., 2010; Kinman, 2016; Parlangeli et al., 2017), and even higher than compared to other workers (Kinman, 2014). Different organizational factors—often relational factors such as support from superiors, the level of cohesion of one's research group (De Jong et al., 2016; Klassen et al., 2010), and organizational culture (Kinman, 2014)—have been identified as reasons for the high levels of stress highlighted. Among these, the high stress levels of faculty members have been related to work-life balance issues, which might have increased during the lockdown period. An Australian study had shown that university professors' perceptions of work-associated stress were related to poor work-life balance, causing conflicting situations between these two life spheres (Bell et al., 2012). Other studies have focused on university careers in the medical field showing how the high operational demands can induce high levels of stress which, in turn, in many cases can lead to career abandonment (Shanafelt et al., 2009). This causal chain, which from a failure to balance personal and work life, leads to a loss of psychological and physical well-being, up to the abandonment of one's profession, is even more evident for women who have entered university careers in the medical field (Isaac et al., 2014).

Hence, due to the special conditions established to deal with the spread of the pandemic, we believe that this population deserves specific attention. In fact, following the spread of the pandemic emergency, some studies have tried to evaluate the consequences that the transition from an activity in presence to one at a distance, and in conditions of isolation, could have for academics (Ammar et al., 2020; Besser et al., 2020). A recent study investigated the stress levels of surgeons at five US teaching hospitals. A total of 337 surgeons, who also served as faculty, participated in the study. The results showed high levels of stress in all participants. In addition, it was found that the stress levels of female surgeons were significantly higher than those of their male colleagues (Mavroudis et al., 2021).

Many questions, however, still remain unanswered. For example, it is necessary to evaluate whether the likely increase in stress levels of faculty members in periods of home quarantine has impacts on their relationships with colleagues and students. Above all, it is relevant to determine what are the behavioral consequences, in connection with psychological distress, with reference to research and teaching activities. A study conducted before the spread of the pandemic on Italian university precarious workers revealed how when the perception of stress increases also the perception of violations related to behaviors that should ensure the integrity of research increases (Parlangeli et al., 2020). Moreover, the results of this study showed that the relationship between stress and misconduct was more accurately understood when socio-technical factors such as lack of adequate support from supervisors and lack of relationships with colleagues were considered. This is a relevant issue since those relationships are likely to be hindered during a lockdown period, and more difficult to maintain. Therefore the condition of isolation experienced by academics, in addition to psychological symptoms, may also have consequences on the perception and enactment of behaviors that can be qualified as misconduct able to negatively affect research integrity (Bouter et al., 2016; Fanelli, 2009; Steneck, 2006) or unethical behaviors (Davis et al., 2007; DuBois et al., 2012, 2013; Kish-Gephart et al., 2010).

## The Study

The study presented here was carried out with the main objective of shedding light on aspects related to stress, its causes and its consequences, in a sample of Italian faculty members during the first lockdown period due to the Covid-19 pandemic outbreak—from mid March to end of May 2020. More specifically, we sought to assess:

- What were the housing conditions of the participants during the lockdown period, what were the main issues related to academic activity, and what, if any, were the relationships between these variables;
- The conditions of stress, well-being, work-life balance and the relationships between these variables.

The hypotheses that motivated the study were related to the possibility that, along with variables such as age and gender (Parlangeli et al., 2017; Isaac et al., 2014), stress levels would increase and well-being levels would decrease, particularly as a consequence of the occurrence of unbalanced work-life conditions (hypothesis 1—H1). Moreover, it was hypothesized that as stress levels increased and dysfunctional work-life relationships occurred, both perceptions of the frequency of misconduct and perceptions of their increase, during home quarantine, would increase (hypothesis 2—H2).

### Participants and Procedure

The study involved 2335 university teachers working in three Universities in Tuscany (Firenze, Pisa, Siena), which were contacted individually via their institutional e-mail. A total of 581 (24.88%) responded to the survey and were included in the analyses reported below. In the contact e-mail participants were asked to fill in a self-reported online questionnaire that took about 15–20 min to be completed. The letter of invitation explained the purpose of the study, making them clear that their participation would be voluntary and anonymous, and that they could freely stop filling-in the questionnaire at any time.

The introduction to the questionnaire served as informed consent, and also let the participants know that the research had been approved by the Ethics Committee for Research in the Human and Social Sciences of the University of Siena (CAREUS) (act no. 6/2020).

The survey was launched online in October 2020 on the Google Forms platform, and the data were collected over a period of 3 months (October–December 2020).

### Materials and Methods

The questionnaire consisted of six sections (the entire questionnaire is provided as Supplementary Material 1). The first section comprised nine questions and was aimed at collecting socio-demographic information such as gender, age, position at the university, seniority and scientific research area of each participant.

The following three sections, all constructed specifically for this study, had questions related to working and living conditions during the first lockdown period in March–May 2020. The second section investigated distance work settings by asking five questions on the house characteristics (“How do you evaluate the environment you lived in during the quarantine period-mid March/end of May 2020-? wide, uncrowded, well located, well equipped, pleasant”). Answers were collected on a five-point Likert scale. The third section investigated the level of adequacy of technological equipment. It consisted of 4 questions such as “During this time of distance working, did you have access to a computer (or a tablet) for your work activity” with a YES/NO answer. A further question asked participants to evaluate on a five-point Likert scale whether the Internet connection was adequate for their needs.

The fourth section was structured to collect information on problems experienced by respondents during lockdown in academic work activities. Eight different dimensions of academic work life have been considered: online exams, relationships with professors, online lectures, relationships with students, administrative practices, receiving technical/administrative support, research activities, and writing papers. For each dimension, participants were asked to rate how problematic it had been on a five-point Likert scale, from 0 (not at all) to 4 (very much), during lockdown. To avoid response bias, responses to the questions in the previous sections were collected on scales in which the positive and negative poles were not always the same.

The fifth section included three standardized scales that were aimed at measuring, respectively, the level of perceived stress, the degree of psychological well-being, and the level of interference between work and private life. Perceived stress was measured by the short version of the Perceived Stress Scale (PSS) (Cohen & Williamson, 1988; Cohen et al., 1983; Warttig et al., 2013). This scale is made up of 4 items that measure the feeling of stress experienced in a particular period. The responses are expressed on a five-point Likert scale varying from 0 = “never” to 4 = “very often” and the total score is obtained summing the scores for all the items (range: 0–16). The validation study of the Italian four items version of the scale (Mondo et al., 2021) indicates that it has a lower value of Cronbach’s alpha (0.57) than the original ten items version. This can be explained by the presence of only four items, since Cronbach's alpha tends to increase with the number of items (Pedhazur & Schmelkin, 1991), but the shorter version is more suitable when a brief instrument is needed. For the measurement of psychological well-being, the Italian version of the Warwick-Edinburgh Mental Well-Being Scale (WEMWBS) was adopted. This tool consists of 12 items on a five-point response scale (1 = never, 5 = always), and it has been developed (Gremigni & Stewart-Brown, 2011; Tennant et al., 2007) to evaluate a broad concept of well-being understood as positive mental health, which includes affective, cognitive and good psychological functioning aspects. The questionnaire has a limited number of items, to facilitate administration to the general population, and reports only positive aspects of mental health, without however being characterized by a ceiling effect or distortion linked to social desirability. A Cronbach alpha of 0.87 and a test–retest of 0.80 per week indicate good reliability both in terms of internal consistency and stability over time of the Italian version of the scale. The total score is computed by summing the scores for all the items (range: 12–60). The aspects relating to the relationships, functional or dysfunctional, between private and working life, were finally investigated through the questionnaire structured by Fisher and colleagues (Fisher et al., 2009), that was translated into Italian and that we adopted in a previous study (Parlangeli et al., 2020). This scale comprises 17 items that measure 4 factors, having acceptable-to-excellent internal consistency, and respectively related to: Work Interference with Private Life (WIPL, five items, α = 0.94), Work Enhancement of Private Life (WEPL, three items, α = 0.82), Private Life Interference with Work (PLIW, six items, α = 0.92) and Private Life Enhancement of Work (PLEW, three items, α = 0.76). Each item is rated on a five-point Likert scale ranging from 1 = never to 5 = always, and the score for each scale is computed by averaging the scores of the relative items. A confirmatory factor analysis has shown that the Italian translation has the same 4-factor structure as the original English version, with a very good fit (Chi^2^ (113, *N* = 547) = 626.4, NFI = 0.99, NNFI = 99, and CFI = 0.99) to the data (see Supplementary Material 2 for the full details about the scale).

The sixth and last section of the questionnaire was structured to investigate the perception of respondents about the frequency of some misconducts regarding working activities and relationships in the academic environment (Parlangeli et al., 2017, 2020). It included four questions about (a) misconduct by colleagues in research activities, (b) misbehaviors between colleagues, (c) toward students and (d) from students. Some examples of questions are the following: “How often (in your Academic career) did your colleagues engage in incorrect behaviour (e.g., using pieces of other researchers publications, manipulating research data in a confirmatory sense, unduly adding names to publications etc.)?” or “How often do professors behave incorrectly towards students (e.g., not supporting their dissertation paper in thesis commission, being partial during the exams, not providing them with adequate explanations and assistance, not being punctual in conducting the lessons, not be present at the office hours etc.)?”. Answers to these four questions were collected on a five-point Likert scale ranging from 1 = never to 5 = always. Four additional questions investigated whether the perceived frequency of occurrence of these same misbehaviors had changed (increased or decreased) in the quarantine period. For these questions answers ranged from 1 = much decreased to 5 = much increased. These questions were constructed specifically for this study, based on previous analyses already conducted on the populations of Italian faculty (Parlangeli et al., 2017, 2020).

### Statistical Analyses

Descriptive statistics (means, standard deviations and frequencies) were initially computed on the responses to all the items included in the questionnaire, in order to understand the socio-demographic features of the sample, characterize the environment in which participants had spent the lockdown period, and the issues that they experienced in academic work activities.

Correlations among the ratings of the issues and the features of the environment where participants spent lockdown were computed, adjusting *p*-value for multiple comparisons using Holm's method (Holm, 1979). A MANOVA was used to compare male and female participants on the levels of stress, mental well-being and work-life interference simultaneously, following up the multivariate test with univariate ANOVAs. We also computed the correlations between the measures of stress, well-being and work-life balance, and among these variables and the other measures related to living conditions and work issues during lockdown.

Finally, to test the association between stress, work-life interference measures and (a) the perceived frequency of misconducts and (b) the perceived change in frequency of misconducts during lockdown, several different ordinal multiple regression logistic models were fitted to the data. These analyses included gender, age, perceived stress and work interference on personal life as predictors of the responses concerning misconducts frequency or misconducts frequency change.

All the statistical analyses were conducted using the statistical software R (v.4.0.2).

## Results

### Participants

The mean age of participants was 49.9 years (*SD* = 10.7), and 48.8% were females. In the following Table 1 are presented the general features of the sample. The majority of respondents were Associate (36.3%) or Full Professor (18.9%). Almost all the respondents (97%) reported to work full time, and had a considerable seniority in academia (summing the time working in any role they had ever filled) (M = 18.6 y, *SD* = 10.5).

**Table 1:** Demographic characteristics of the participants

Variable	*N* (%)	*N*
Age (years) M (SD)	49.9 (10.7)	581
Age		
≤ 30	15 (2.6%)	
31–40	119 (20.5%)	
41–50	160 (27.5%)	
≥ 51	287 (49.4%)	
Gender = Female	279 (48.8%)	572
Academic position		578
Assistant Professor/Research Fellow	111 (19.2%)	
Associate Professor	210 (36.3%)	
Fixed-term Researcher	42 (7.27%)	
Full Professor	109 (18.9%)	
Lecturer	8 (1.38%)	
Post-Doctoral Fellow	98 (17.0%)	
Seniority in Academia (years) M (SD)	18.6 (10.5)	561
Seniority in current university		571
0–5 years	167 (29.2%)	
6–15 years	144 (25.2%)	
16–25 years	166 (29.1%)	
26 or more years	94 (16.5%)	
Seniority in current position (years) M (SD)	8.16 (8.13)	569
Time regime = Full time	559 (97.0%)	576
Scientific area		454
Agricultural and Veterinary Sciences	44 (9.69%)	
Architecture and Engineering	54 (11.9%)	
Economics, Statistical, Political and Social Sciences	64 (14.1%)	
Humanities, Pedagogical and Psychological Sciences	125 (27.5%)	
Mathematics, Physics, Chemistry and Geology	109 (24.0%)	
Medical Sciences	58 (12.8%)	

### Information About the Lockdown Period (March–May 2020)

In Table 2 are reported descriptive statistics about the ratings of the environment where respondents spent lockdown along different dimensions. As it can be seen, for all the dimensions considered the average scores were significantly above the midpoint of the scale (3), indicating that in general the environment was positively evaluated. The household features that had the lower average ratings (less positive evaluations) concerned the wideness of the environment, and the extent it was not crowded. 19.6% of the respondents, however, reported that the environment lacked the equipment they needed (*n* = 113).

**Table 2:** Average scores for the ratings of the environment/household where lockdown was spent along different dimensions

Dimension	Mean	SD	SE	Range	95% CI
Wide (vs narrow)	3.8	1.1	0.05	4 (1–5)	3.7–3.8
Uncrowded (vs crowed)	3.7	1.1	0.04	4 (1–5)	3.7–3.8
Well located (vs poorly located)	4.3	0.9	0.04	4 (1–5)	4.3–4.3
Well equipped (vs poorly equipped)	4.0	1.3	0.1	4 (1–5)	3.9–4.1
Pleasant (vs unpleasant)	4.1	1.0	0.04	4 (1–5)	4.1–4.2

All but three respondents had access to a PC or a tablet during lockdown, and almost all of them had exclusive access to it (93.8%) and reported that it was adequate for their needs (95.5%). An internet connection was available in the household in 99.8% of the cases. However, 18.2% of respondents rated the internet connection as not (13.6%) or absolutely not (4.6%) adequate for their needs. The ratings about the inadequacy of the equipment were negatively correlated with the degree the household was considered well-located (*r* = −0.16, *p* < 0.01), and positively with the ratings about the inadequacy of the available internet connection (*r* = 0.36, * p* < 0.001).

### Work and Related Issues

**Fig. 1:**
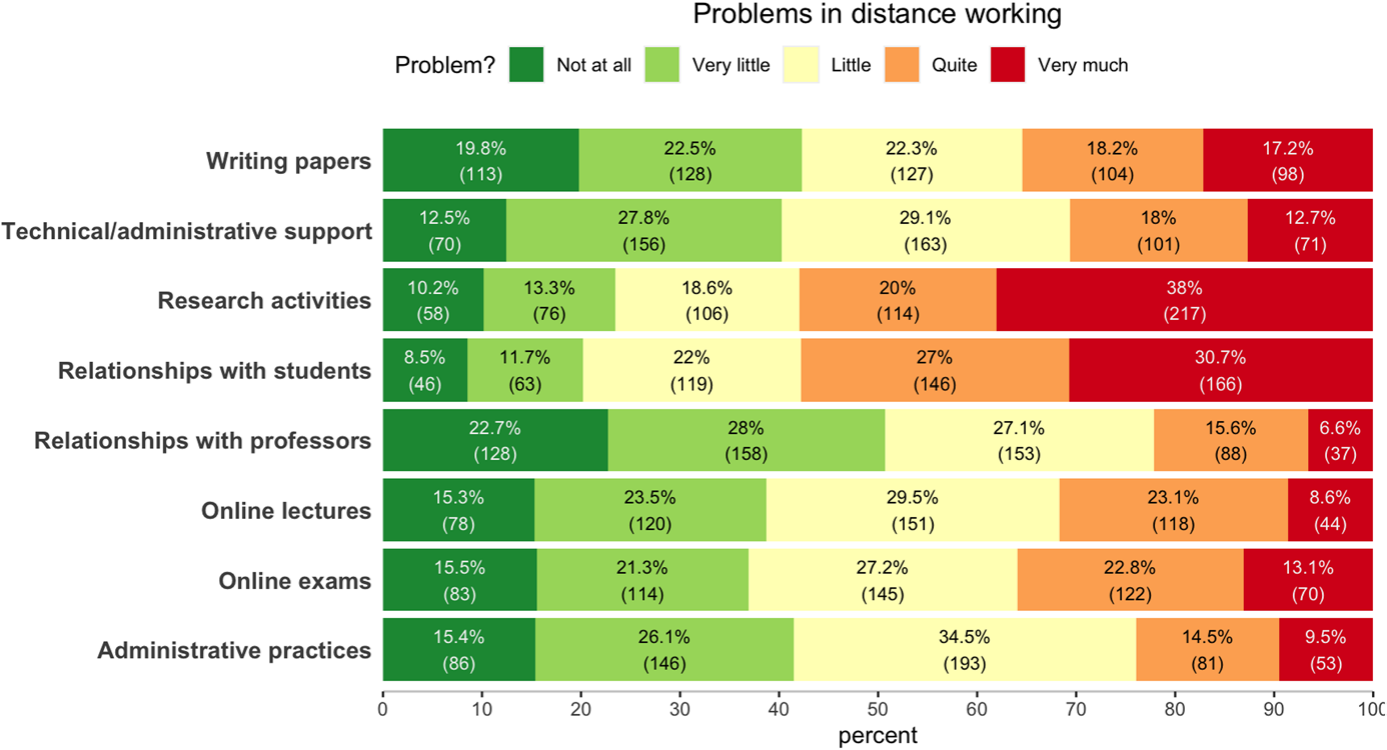
Stacked frequencies bar plots of the responses concerning the problems experienced in learning activities during the lockdown. The length of the bars represents the percentage of respondents that, for each type of problem, selected one of the different response categories (color coded)

Table 3 reports respondents’ ratings of the problems experienced during the lockdown concerning different dimensions of academic work life (expressed on a scale from 0 = ‘not a problem at all’ to 4 = ‘very much of a problem’). The highest ratings, significantly higher than the midpoint of the scale (2) were found for the dimensions concerning the research activities and the relationships with students. The lowest ratings were found concerning the relationships with other colleagues. For this dimension, and for other two ones concerning respectively carrying out administrative duties and giving online lectures, the average ratings were also significantly lower than the midpoint of the scale. For the remaining dimensions the average ratings were not significantly different from the midpoint level. In Fig. 1 are reported the distributions of responses to the survey items related to the issues in academic work life.

**Table 3:** Average scores for the ratings of the difficulties experienced during lockdown concerning different dimensions of academic work life

Variable	Mean	SD	SE	Range	95% CI
Issues with online exams	2.0	1.3	0.1	4 (0–4)	1.9–2.1
Issues in relationships with professors	1.6	1.2	0.05	4 (0–4)	1.5–1.7
Issues in online lectures	1.9	1.2	0.1	4 (0–4)	1.8–2.0
Issues in relationships with students	2.6	1.3	0.1	4 (0–4)	2.5–2.5
Issues administrative practices	1.8	1.2	0.05	4 (0–4)	1.7–1.9
Issues in receiving technical/administrative support	1.9	1.2	0.1	4 (0–4)	1.8–2.0
Issues in research activities	2.6	1.4	0.1	4 (0–4)	2.5–2.7
Issues with writing papers	1.9	1.4	0.1	4 (0–4)	1.8–2.0

The ratings for each dimension were significantly and positively correlated with all the other dimensions, with correlation coefficients ranging between 0.15 and 0.63. The ratings for the inadequacy of the internet connection were significantly correlated with issues in online lectures (*r* = 0.18, *p* < 0.01), in carrying out administrative duties (*r* = 0.14, *p* < 0.05) and in getting online support (*r* = 0.16, *p* < 0.05). The ratings about the inadequacy of the equipment were positively correlated with issues in administrative duties (*r* = 0.16, *p* < 0.05), in getting technical support (*r* =  0.17, *p* < 0.01), in research activities (*r* = 0.21, *p* < 0.001) and in writing papers (*r* = 0.22, *p* < 0.001). No further significant correlations were found between the ratings of the issues considered and the ratings of the features of the household where participants had spent the lockdown period.

### Stress, Mental Well-Being and Work-Life Balance During Lockdown

Table 4 presents the average scores for measures of stress, well-being and interference/enhancement between work and personal life, along with 95% confidence intervals. The average level of perceived stress, as measured with the short version of the Perceived Stress Scale (PSS) (Cohen & Williamson, 1988; Mondo et al., 2021) indicated a level of stress (*M* = 6.3, *SD* = 3.2) higher than previously published Italian averages for individuals of comparable age (*M* = 5 between 41 and 50 y, *M* = 5.8 above 51 years) (Mondo et al., 2021), and higher than UK averages for individuals aged between 45 and 54 year (*M* = 5.69), between 55 and 64 years (*M* = 5.76) and over 64 years (*M* = 5.32) (Warttig et al., 2013). The average level of wellbeing, as measured with the WEMWBS scale (M = 40.4, SD = 8) was instead significantly lower than the published value (42.2) for Italian workers (Gremigni & Stewart-Brown, 2011), overall and in the younger (≤ 30 years) and older age groups (> 57) years). The pattern of means for the scores on the scales measuring interference between work and private life (Fisher et al., 1998) indicates moderate levels of interference from work to private life (*M* = 3.0), not significantly higher than the midpoint of the scale (3), low levels of private life interference with work (*M* = 2.1), and low to moderate levels of enhancement of private life from work (*M* = 2.6) and of work from private life (*M* = 2.9).

**Table 4:** Average scores for the measures of Perceived stress, well-being and study-personal life interference

Variable	Mean	SD	SE	Range	95% CI
Perceived stress (PSS)	6.3	3.2	0.1	16 (0–16)	6.0–6.6
Mental Well-being (WEMWBS)	40.4	8.0	0.3	60 (12–60)	39.7–41.1
Work Interference with Private Life (WIPL)	3.0	1.1	0.04	4 (1–5)	3.0–3.1
Work Enhancement of Private Life (WEPL)	2.6	0.9	0.04	4 (1–5)	2.5–2.7
Private Life Interference with Work (PLIW)	2.1	0.9	0.04	4 (1–5)	2.0–2.5
Private Life Enhancement of Work (PLEW)	2.9	0.9	0.04	4 (1–5)	2.8–3.0

*PSS* Perceived Stress Scale, *WEMWBS* Warwick-Edinburgh Mental Well-Being Scale, *WIPL* Work Interference with Private Life, *WEPL* Work Enhancement of Private Life, *PLIW* Private Life Interference with Work, *PLEW* Private Life Enhancement of Work

To test for differences in the measures of stress, well-being and work-life interferences across groups defined by gender, we conducted a MANOVA using all the measures as dependent variables, and gender as independent variable (Table 5). The results of the multivariate test showed a significant effect of gender [F(6, 549) = 7.88, *p* < 0.001] on the linear combination of the dependent variables. The univariate ANOVAs that we conducted to follow-up the significant MANOVA showed a significant effect of gender on perceived stress [F(1, 554) = 18.6, *p* < 0.001], mental well-being [F(1, 554) = 13.04, *p* < 0.001] and work interference with personal life [F(1, 554) = 34.02, *p* < 0.001]. Female respondents had higher levels of perceived stress (*M* = 6.88, *SD* = 3.36) and interference of work with personal life (*M* = 3.29, SD = 1.04), and lower levels of mental well-being (*M* = 39.1, *SD* = 7.64) than males (PSS: *M* = 5.74, *SD* = 2.89; WIPL: *M* = 2.79, *SD* = 0.99, WEMWBS: *M* = 41.4, *SD* = 8.17).

**Table 5:** Results of the Multivariate Analysis of Variance (MANOVA) on the effect of gender on the combination of perceived stress (PSS), mental well-being (WEMBWS) and work personal life interference (WIPL-PLEW)

Multivariate test			Pillai-Bartlett		F	num Df	den Df	*p* value
Effect								
Gender			0.079		7.876	6	549	<0 .0001

Univariate tests								*p* value
Dependent variable	Effect (Gender)			Residual				
	Df	SS	MS	Df	SS	MS	F	
PSS	1	180.2	180.2	554	5380.1	9.711	18.56	<0.0001
WEMWBS	1	778	777.7	554	33,051	59.66	13.04	< 0.001
WIPL	1	35.1	35.11	554	571.6	1.032	34.02	< 0.0001
WEPL	1	0.23	0.233	554	431.1	0.778	0.3	0.564
PLIW	1	0.46	0.456	554	466.3	0.842	0.541	0.462
PLEW	1	0.59	0.586	554	432.5	0.781	0.751	0.387

In the upper part of the table the results of the multivariate analysis are reported, while in the lower part are reported the univariate ANOVA results for each dependent variable

*PSS* Perceived Stress Scale, *WEMWBS* Warwick-Edinburgh Mental Well-Being Scale, *WIPL* Work Interference with Private Life, *WEPL* Work Enhancement of Private Life, *PLIW* Private Life Interference with Work, *PLEW* Private Life Enhancement of Work

We analysed the correlations among the different measures of stress, well-being and work-life interference. Perceived stress (PSS) was positively correlated with the work-life interference measures (WIPL: *r* = 0.36, *p* < 0.001; PLIW: *r* = 0.41, *p* < 0.001) and negatively correlated with enhancement ones (WEPL: *r* = −0.24, *p* < 0.001; PLEW: *r* = −0.31, *p* < 0.001). Mental well-being was strongly and negatively correlated with perceived stress (*r* = −0.57, *p* < 0.001), and exhibited a pattern of correlations with the work-life interference measures opposite to the one of these variables showed with perceived stress, being negatively correlated with interference variables (WIPL: *r* = −0.24, *p* < 0.001; PLIW: *r* = −0.31, *p* < 0.001) and positively correlated with enhancement ones (WEPL: *r* = 0.32, *p* < 0.001; PLEW: *r* = 0.42, *p* < 0.001). Work-life interference measures were also moderately correlated among each other in a very regular pattern: interference variables were positively correlated among each other, and so were enhancement ones. Enhancement measures were then negatively correlated with interference ones.

**Fig. 2:**
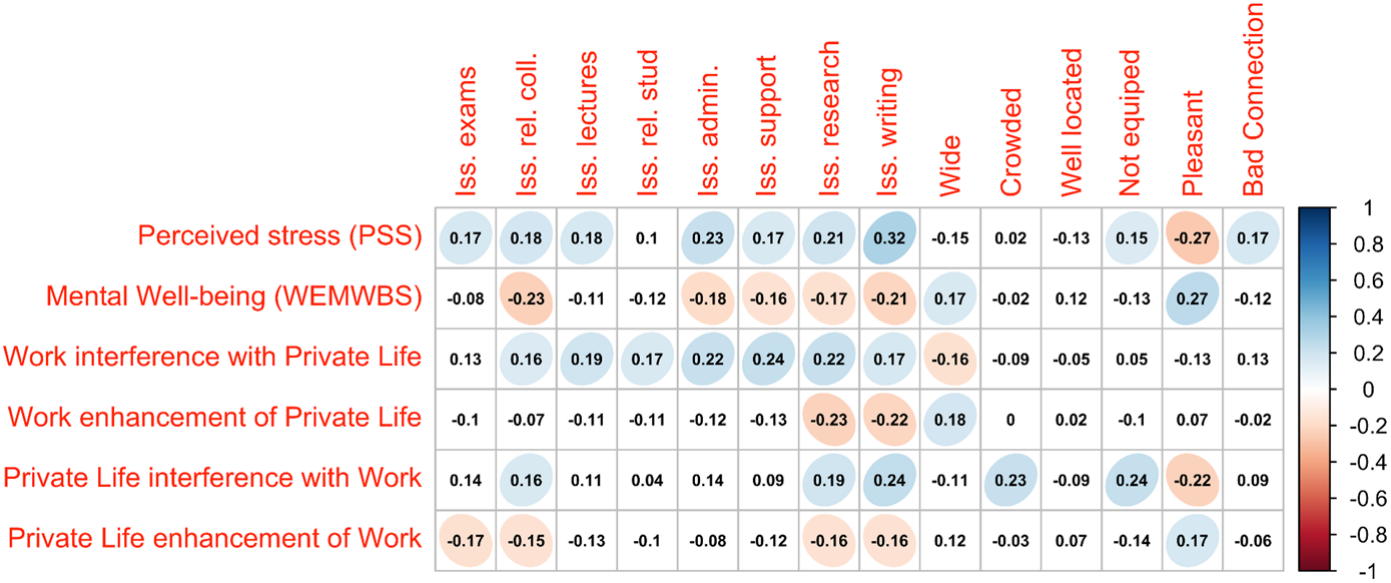
Plot of the correlations among stress, well-being and WLB measures, issues in academic life and features of the household where respondents spent lockdown. The coefficients reported are Pearson’s product momentum indices, and statistically significant correlations are represented by colored ovals

In Fig. 2 are plotted the correlations between the stress, well-being and work-life interference variables, and the other variables related to living and working during lockdown (the significant correlations are displayed as colored ovals). Perceived stress was positively correlated with all issues in all domains except the one concerning the relationships with students. The strongest correlation was with issues in writing papers (*r* = 0.32, *p* < 0.001), followed by issues in carrying out administrative duties (*r* = 0.23, *p* < 0.001) and issues in research activities (*r* = 0.21, *p* < 0.001). Perceived stress was also positively correlated with the ratings of the inadequacy of the equipment of the locations for the respondents’ needs (*r* = 0.15, *p* < 0.001) and of the inadequacy of the available internet connection (*r* = 0.17, p < 0.001), and it was negatively correlated with the ratings of pleasantness (*r* = −0.27, *p* < 0.001) of the household where the lockdown was spent. Mental well-being was negatively correlated with the following issues: relationships with colleagues (*r* = −0.23, *p* < 0.001), writing papers (*r* = −0.21, *p* < 0.001), carrying out administrative duties (*r* = −0.18, *p* < 0.01), conducting research activities (*r* = 0.17, *p* < 0.05), and getting technical/administrative support online (*r* = −0.16, *p* < 0.05). It was instead positively correlated with the ratings of pleasantness (*r* = 0.27*, p* < 0.001) and wideness (*r* = 0.27, *p* < 0.01) of the location where respondents spent lockdown.

### Misconducts in Academic Work Life

**Fig. 3:**
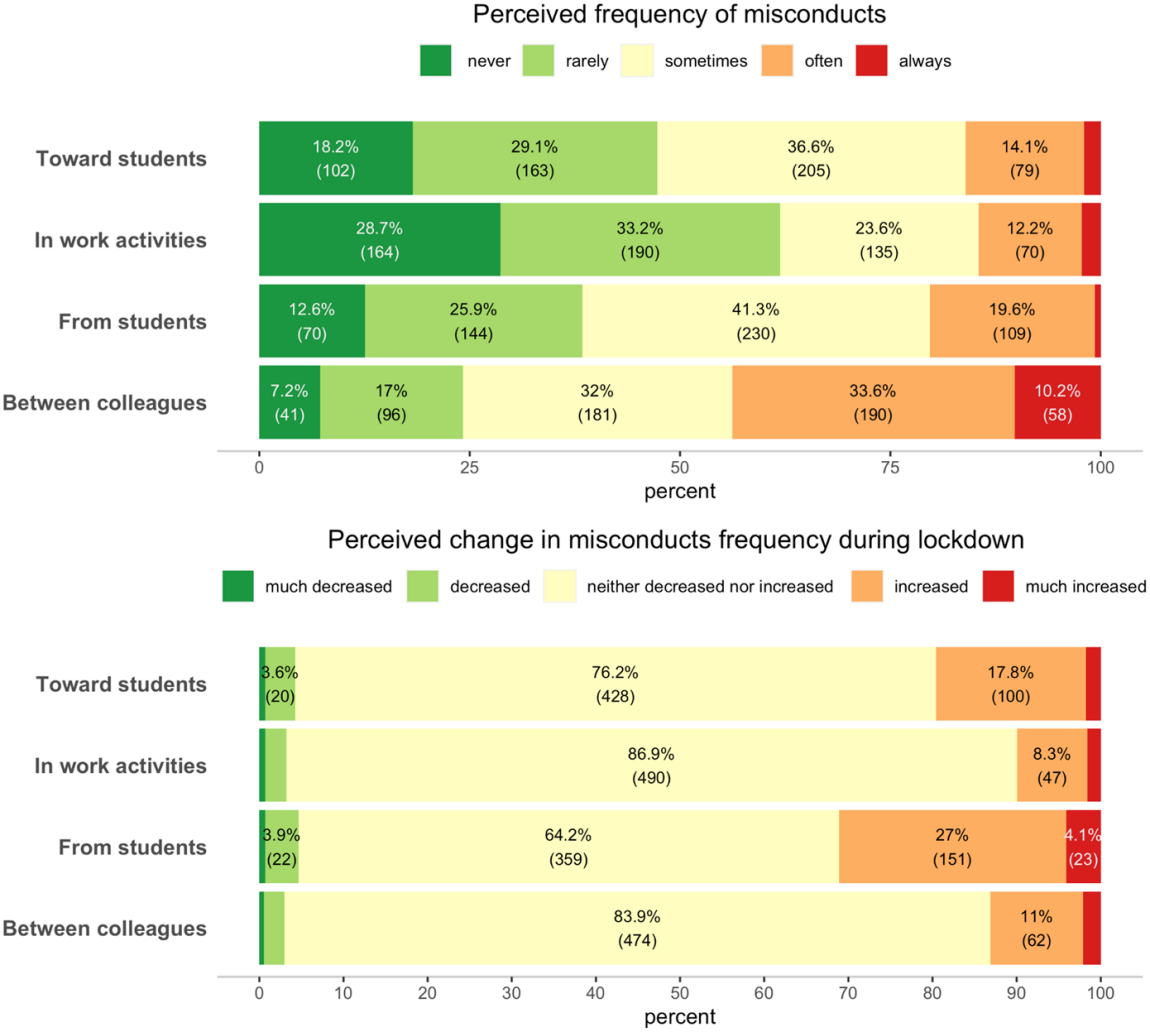
Stacked frequencies bar plots of the distribution of responses to the items related to the frequency of different types of misconduct in academia (top), and to their frequency change during the lockdown period (bottom). In the top plot orange and red bars indicate the percentage of respondents that reported the highest frequency of misconduct (often or always), while in the bottom plot orange and red bars indicate the percentage of respondents that reported that the frequency of misconduct had increased during the lockdown period

Figure 3 represents participants’ responses concerning different types of misconduct in academia (top plot) and the perceived change in misconduct frequency during lockdown (bottom plot). The perceived frequency of the different types of misconduct varies across types, with misconduct in relationships with colleagues and from students that were judged as more frequent. The least frequent misconduct were judged to be those in working activities. The majority of respondents believed that the frequency of the different misconduct had not changed during lockdown, but for all the misconduct more participants reported that the frequency had increased during lockdown than that it had decreased. Misconducts from students were judged to have increased by about one third of participants.

**Fig. 4:**
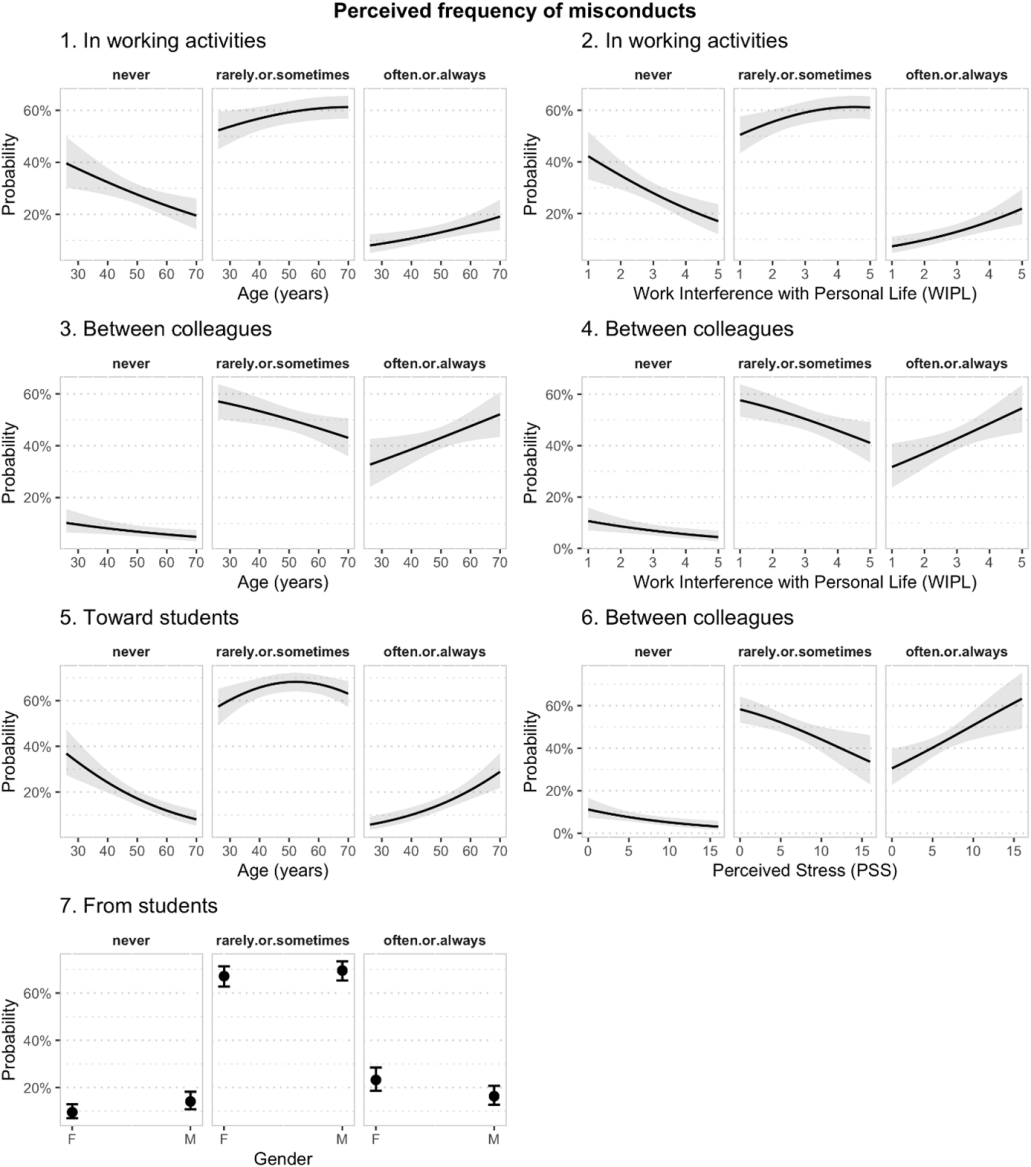
Predicted probabilities for the response categories relative to the frequency of different misconducts in academia as a function of age (1, 3, 5), work interference with personal life (2, 4), gender (7) and perceived stress (6)

To analyse the association between the perception of the frequency of misconducts, perceived stress (PSS) and work-personal life interference (WIPL), we used ordinal logistic regression models. The judgments about the frequency of the different types of misconducts were first recoded on three levels: “never”, “rarely or sometimes” and “often or always”. For each type of misconduct we then used the recoded variable as dependent variable in the regression, including as predictors gender, age, perceived stress as measured by the perceived stress scale (PSS), and interference between work and personal life (WIPL). Numerical predictors were centered on their mean. In Table 5 are reported the results of the analyses for all the types of misconduct considered, with the predictors’ coefficients reported as Odd Ratio (OR). The age of respondents was significantly associated with the perceived frequency of misconducts in working activities (*p* < 0.01), between colleagues (*p* < 0.05) and toward students (*p* < 0.001). The OR for age were higher than 1, indicating that older respondents were more likely to consider the misconducts as frequent, as it can be also seen in the plots of the response probabilities in Fig. 4. Gender was only associated with misconducts from students, which were considered less frequent by male respondents than by female ones. The level of interference between work and personal life was also significantly associated with the perceived frequency of misconduct in working activities (*p* < 0.001) and between colleagues (*p* < 0.01), which in turn was also associated with perceived stress (*p* < 0.01). As it can be seen in the plots in Fig. 4, both perceived stress and interference between work and personal life tended to increase the likelihood of participants reporting that misconducts were common in academia.

The plots also reveal that for misconduct in working activities both the probability of participants reporting that the misconducts happen “rarely or sometimes” and the probability that they happen “often or always” increased with age and WIPL. For misconducts between colleagues, instead, only the likelihood of participants reporting that the misconducts happen “often or always” tended to increase with the independent variables, while the probability that they happen rarely or sometimes decreased. This reflects the differences between the misconducts in how frequently they were judged to happen “rarely or sometimes”.

**Fig. 5:**
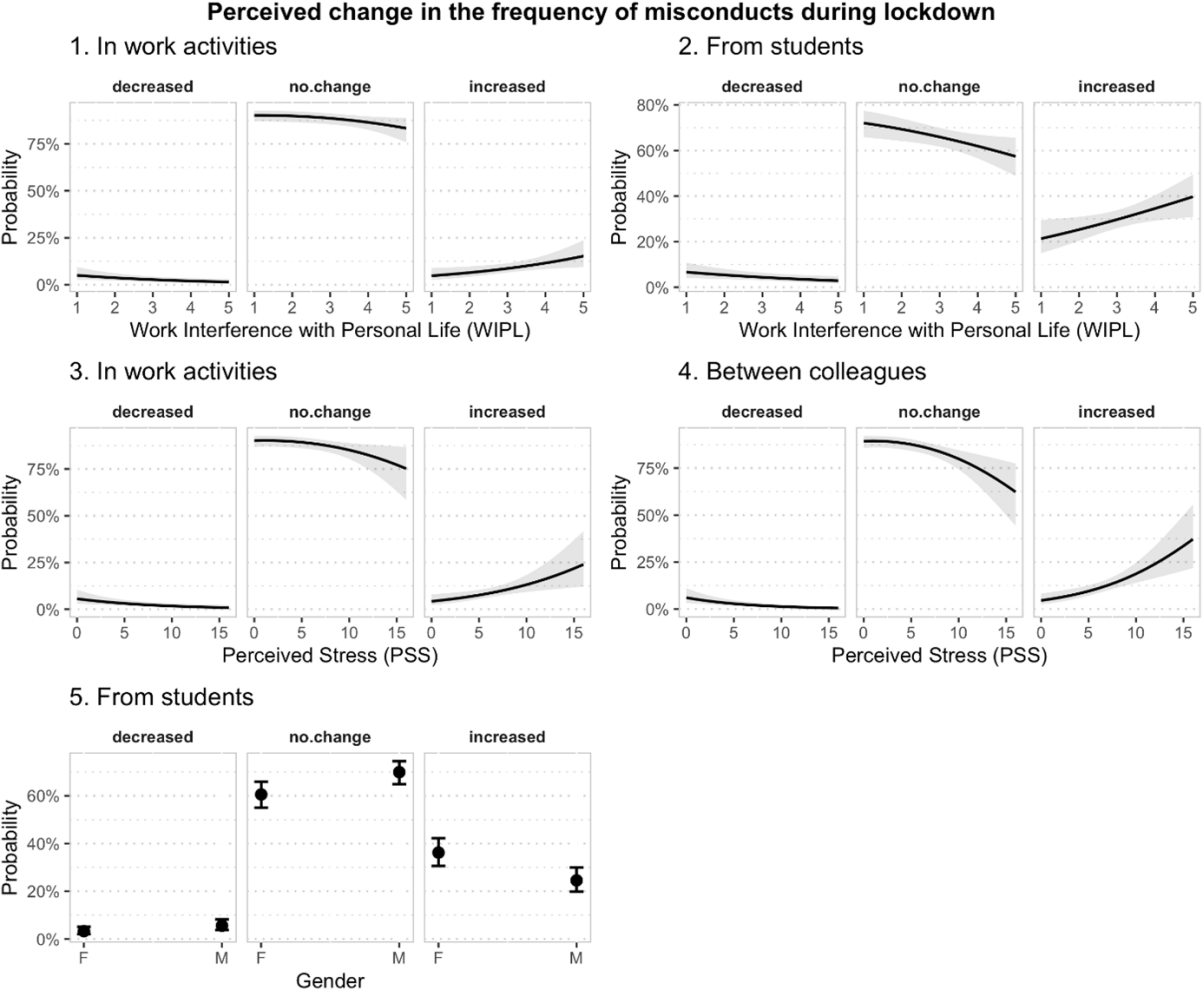
Predicted probabilities for the response categories relative to the perception of the change of the frequency of different misconducts in academia during lockdown, as a function of work interference with personal life (top row), perceived stress (middle row), and gender (bottom row)

The same type of statistical analysis was used to model the perceived *change* of the frequency of the different misconducts during the lockdown period. The responses were first recoded into three levels: “decreased”, “no change” and “increased”, and the recoded variables were used as dependent variables using the same predictors as in the previously reported analysis. The results, presented in Table 6 and Fig. 5, showed that the perceived change in the frequency of misconduct was not associated with the age of respondents. Higher levels of perceived stress were significantly associated with higher likelihood of participants reporting an increase in the frequency of misconduct in working activities (*p* < 0.01) and between colleagues (*p* < 0.001) during lockdown (as opposed to a decrease or to no change). Higher levels of interference between work and personal life were also associated with higher probability of participants reporting increases in the frequency of misconducts in work activities (*p* < 0.05) and from students (*p* < 0.05). Finally, female participants were more likely than males to report that the frequency of misconduct from students during lockdown had increased (*p* < 0.01).

**Table 6:** Results of the Ordinal regression model of the perceived change in the frequencies of different misconducts during lockdown. In each model, the ordinal response about the perceived change in frequency of a different type of misconduct (in working activities, between colleagues, toward students, from students, measured on 3 levels: decreased, neither increased nor decreased, increased) was regressed onto 4 predictors: gender, age, perceived stress (PSS) and work interference with personal life (WIPL)

	In working activities			Between colleagues			Toward student			From students		
Predictors	OR	CI	*p*	OR	CI	*p*	OR	CI	*p*	OR	CI	*p*
Gender (= M)	0.85	0.50–1.43	0.549	0.68	0.41–1.11	0.125	0.78	0.52–1.17	0.234	0.57	0.40–0.83	**0.003**
Age	0.99	0.96–1.01	0.241	0.98	0.96–1.01	0.154	1.00	0.98–1.02	0.898	0.99	0.97–1.00	0.120
PSS	1.13	1.04–1.23	**0.005**	1.17	1.08–1.27	**< 0.001**	0.97	0.90–1.03	0.337	1.02	0.96–1.09	0.469
WIPL	1.38	1.06–1.80	**0.017**	1.15	0.91–1.47	0.243	1.03	0.84–1.27	0.756	1.25	1.04–1.50	**0.018**
Obs	554			555			553			550		
Deviance	490.386			537.733			728.056			841.914		

*OR* Odd Ratio, *CI* Confidence Intervals

In the table, for each model are presented the Odd Ratio (OR) estimated from the analysis of the four predictor variables included in the model, along with 95% confidence intervals for the OR and *p* value

## Discussion

The study presented here was conducted with the aim of exploring how living and working conditions during the first period of isolation due to Covid-19 in Italy may have affected the psychophysical well-being of faculty and their perceptions of misconduct in academia.

The results appear relevant from several perspectives.

First, a relationship between the adequacy of the available technology and the issues reported in work practice has been highlighted, this especially in reference to the adequacy of the internet connection (H1). This is evident in the results showing that nearly 20% of respondents report not having adequate equipment and complain of poor internet connection. Interestingly, internet connection difficulties and inadequate available technology correlated with a home location judged unfavorable. This suggests that even workers such as faculty members who are usually equipped with advanced technology are at risk of experiencing digital divide when operational needs become substantially based on internet connection, as during a period of home quarantine (Philipsen et al., 2019; Singh et al., 2021).

The measures used to assess the levels of stress (Cohen & Williamson, 1988; Cohen et al., 1983; Warttig et al., 2013) and psychophysical well-being (Cohen & Williamson, 1988; Cohen et al., 1983; Warttig et al., 2013) are all consistent, i.e. as one increases, the other decreases. This also occurs for the measures relating to interference and enhancement between work and private life (Fisher et al., 2009). Above all, these data make evident a general condition of distress that, although not exceedingly high, is significantly higher than the benchmarks. This condition is more problematic for female teachers who report higher levels of stress and are likely to suffer greater work-life imbalance. This can be ascribed to a gender issue that has already emerged in the literature for female professors in medical sciences who also perform caring activities (Mavroudis et al., 2021). In this case, however, it is also found for female university professors in non-medical disciplines, and probably points to home behavioral customs that expect a centrality of the female component.

Finally, our data suggest that higher stress levels may facilitate the perception of misconduct in the academic setting, similarly to what was found by Parlangeli and colleagues (Parlangeli et al., 2017) with different scales and with a smaller sample (H2). This is found both when assessing perceptions of stress (PSS) as when highlighting negative work-life interference outcomes (WIPL). Beside statistical significance, the size of the estimated effect of work interference with private life (OR between 1.27 and 1.37) is not trivial, as it means that an increase of one unit in WIPL increases by up to 37% the likelihood of participants reporting misconduct occurring in research activities and between colleagues (as opposed to never occurring). And in our sample about 35% of participants reported quite high levels of work interference with private life. The perceived frequency of misconduct between colleagues was also quite high, as 44% of participants reported they happened often or always. In this regard, it should be emphasized that with increasing age, participating university teachers perceive their working context as an environment where misconduct occurs more frequently, in relation to activities in general, among colleagues, and in relation to students. This result, though deserving future exploration, is likely determined by work experience in the academy that has lasted for a longer time.

Female professors, on the other hand, were more likely than male ones to judge misconduct by students as more frequent. This latter finding is also found in the ratings related to the increased occurrence of misconduct from students during the home quarantine, and again points to a gender issue that is likely to arise from conditions of domestic activities imbalance in the family environment.

## Limitations

The study presented here has some limitations. First, the faculty members were contacted some months after experiencing the period of isolation which the questionnaire referred to. This may have led to both a loss of memories and their alteration. And it is difficult to determine if and how much this happened on the basis of the data collected.

In order to avoid the administration of an overly long questionnaire, many of the questions were formulated in a rather general manner. This happened especially with reference to problems and, to an even greater extent, with misconduct. Therefore, on the basis of the results obtained, it is difficult to relate stress levels to specific behaviors or to say which is the most frequent misconduct.

Finally, it should be noted that the study did not include any direct measures of misconduct. Therefore, it is difficult to say if perceptions of the frequency of misbehavior, as well as perceptions of their increase during periods of isolation, might correspond to actual increases in misbehavior. While previous studies might suggest that the actual occurrence of misconduct has one of its antecedents in the perception of an unethical organizational climate (Davis, 2003; Davis et al., 2007; Martinson et al., 2010), our study might add to this picture and might provide incentives for follow up work.

## Conclusions

The findings obtained are complex and probably involve mutually affecting variables. The adequacy of the working equipment, stress levels, family life intersecting with work life, are all conditions that can reasonably be expected to influence each other. It appears important, however, to have detected how increased stress levels may result in increased perceptions of the frequencies of misbehavior in the academy, and how these frequencies can be seen to increase further in periods of isolation. Increased perceptions of the frequency of misbehavior may in fact be related to increased enactment of misbehavior (Davis et al., 2007; Martinson et al., 2010). Therefore, the integrity of research can be undermined by conditions that foster uncomfortable working conditions, increased stress levels and perceptions of misconduct.

For these reasons it seemed relevant to take into account changes in working conditions resulting from measures aimed at containing the Covid-19 pandemic. Several studies have considered socio-organizational factors as antecedents to the occurrence of behaviors that undermine the integrity of research in the academy (Kish-Gephart et al., 2010; Martinson et al., 2013; Redman & Caplan, 2017). Research, however, could not have foreseen the dramatic changes in ordinary academic activity that have occurred in many countries due to the implementation of preventive measures to contain the spread of the Covid-19 pandemic. New analyses, aimed at evaluating these new working settings, have therefore been deemed necessary (Johnson et al., 2020; Watermeyer et al., 2021). Focusing on stress and misconduct, this study found that the organization of working at home, when implemented as an emergency and without proper planning, and particularly with regard to female teachers, is likely to have negative consequences for the psychological and physical well-being of faculty and for integrity of research.

## Supplementary Information

Below is the link to the electronic supplementary material.

Supplementary file1 (DOCX 21 kb)

Supplementary file2 (DOCX 18 kb)

## References

[CR1] Ammar N, Aly NM, Folayan MO, Khader Y, Virtanen JI, Al-Batayneh OB, Mohebbi SZ, Attia S, Howaldt HP, Boettger S, Maharani DA, Rahardjo A, Khan I, Madi M, Rashwan M, Pavlic V, Cicmil S, Choi YH, Joury E (2020). Behavior change due to COVID-19 among dental academics—The theory of planned behavior: Stresses, worries, training, and pandemic severity. PLoS ONE.

[CR4] Bell A, Rajendran D, Theiler S (2012). Job stress, wellbeing, work-life balance and work-life conflict among Australian academics. E-Journal of Applied Psychology.

[CR5] Besser, A., Lotem, S., & Zeigler-Hill, V. (2020). Psychological stress and vocal symptoms among university professors in israel: Implications of the shift to online synchronous teaching during the COVID-19 pandemic. *Journal of Voice* (in Press). 10.1016/j.jvoice.2020.05.028 10.1016/j.jvoice.2020.05.028 PMC7274605 32600872

[CR6] Bouter, L. M., Tijdink, J., Axelsen, N., Martinson, B. C., & Ter Riet, G. (2016). Ranking major and minor research misbehaviors: Results from a survey among participants of four world conferences on research integrity. *Research Integrity and Peer Review,**1*, 17. 10.1186/s41073-016-0024-5 10.1186/s41073-016-0024-5 PMC5803629 29451551

[CR7] Cao W, Fang Z, Hou G, Xu X, Dang J, Zheng J (2020). The psychological impact of the COVID-19 epidemic on college students in China. Psychiatry Research.

[CR8] Catano VM, Francis L, Haines T, Kirpalani H, Shannon H, Stringer B, Lozanski L (2010). Occupational stress in Canadian universities: A national survey. International Journal of Stress Management.

[CR9] Cohen S, Kamarck T, Mermelstein R (1983). A global measure of perceived stress. Journal of Health and Social Behavior.

[CR10] Cohen, S., & Williamson, G. (1988). Perceived stress in a probability sample of the U.S.. In S. Spacapam & S. Oskamp (Eds.) *The social psychology of health: Claremont symposium on applied social psychology* (pp. 31–67) Sage.

[CR12] Davis MS (2003). The role of culture in research misconduct. Accountability in Research.

[CR13] Davis MS, Riske-Morris M, Diaz SR (2007). Causal factors implicated in research misconduct: Evidence from ORI case files. Science and Engineering Ethics.

[CR14] De Jong T, Wiezer N, De Weerd M, Nielsen K, Mattila-Holappa P, Mockałło Z (2016). The impact of restructuring on employee well-being: A systematic review of longitudinal studies. Work & Stress.

[CR15] DuBois JM, Kraus E, Vasher M (2012). The development of a taxonomy of wrongdoing in medical practice and research. American Journal of Preventive Medicine.

[CR16] DuBois JM, Anderson EE, Chibnall J, Carroll K, Gibb T, Ogbuka C, Rubbelke T (2013). Understanding research misconduct: A comparative analysis of 120 cases of professional wrongdoing. Accountability in Research.

[CR17] Fanelli D (2009). How many scientists fabricate and falsify research? A systematic review and meta-analysis of survey data. PLoS ONE.

[CR18] Fiorillo A, Gorwood P (2020). The consequences of the COVID-19 pandemic on mental health and implications for clinical practice. European Psychiatry.

[CR19] Fisher, G. G., Bulger, C. A., & Smith, C. S. (2009). Beyond work and family: A measure of work/non work interference and enhancement. *Journal of Occupational Health Psychology,**14*(4), 441–456. 10.1037/a0016737 10.1037/a0016737 19839663

[CR20] Galea S, Merchant R, Lurie N (2020). The mental health consequences of COVID-19 and physical distancing: The need for prevention and early intervention [published online April 10th, 2020]. JAMA Internal Medicine.

[CR21] Gremigni P, Stewart-Brown S (2011). Una misura del benessere mentale: Validazione italiana della Warwick-Edinburgh Mental Well-Being Scale (WEMWBS). Giornale Italiano di Psicologia.

[CR22] Gualano, M. R., Lo Moro, G., Voglino, G., Bert, F., & Siliquini, R. (2020). Effects of Covid-19 lockdown on mental health and sleep disturbances in Italy. *International Journal of Environmental Research and Public Health,**17*(13), 4779. 10.3390/ijerph17134779 10.3390/ijerph17134779 PMC7369943 32630821

[CR23] Holm, S. (1979). A simple sequentially rejective multiple test procedure. *Scandinavian Journal of Statistics*, 6(2), pp.65–70. Retrieved May 3, 2021, from http://www.jstor.org/stable/4615733

[CR24] Isaac C, Byars-Winston A, McSorley R, Schultz A, Kaats A, Carnes ML (2014). A qualitative study of work-life choices in academic internal medicine. Advances in Health Science Education.

[CR25] Johnson, N., Veletsianos, G., & Seaman, J. (2020). US faculty and administrators' experiences and approaches in the early weeks of the COVID-19 pandemic. *Online Learning*, 24, 6- 21. 10.24059/olj.v24i2.2285

[CR26] Kinman, G. (2014). Doing more with less? Work and wellbeing in academics. *Somatechnics*, *4*(2), 219–235. 10.3366/soma.2014.0129

[CR27] Kinman G (2016). Effort–reward imbalance and overcommitment in UK academics: Implications for mental health, satisfaction and retention. Journal of Higher Education Policy and Management.

[CR28] Kish-Gephart JJ, Harrison DA, Treviño LK (2010). Bad apples, bad cases, and bad barrels: Meta-analytic evidence about sources of unethical decisions at work. Journal of Applied Psychology.

[CR29] Klassen RM, Usher EL, Bong M (2010). Teachers’ collective efficacy, job satisfaction and job stress in cross-cultural context. The Journal of Experimental Education.

[CR30] Lazzerini M, Putoto G (2020). COVID-19 in Italy: Momentous decisions and many uncertainties. The Lancet Global Health.

[CR31] Martinson BC, Crain AL, De Vries R, Anderson MS (2010). The importance of organizational justice in ensuring research integrity. Journal of Empirical Research on Human Research Ethics.

[CR32] Maugeri G, Castrogiovanni P, Battaglia G, Pippi R, D'Agata V, Palma A, Di Rosa M, Musumeci G (2020). The impact of physical activity on psychological health during Covid-19 pandemic in Italy. Heliyon.

[CR33] Mavroudis CL, Landau S, Brooks E, Bergmark R, Berlin NL, Blumenthal B, Cooper Z, Hwang EK, Lancaster E, Waljee J, Wick E, Yeo H, Wirtalla C, Kelz RR (2021). The relationship between surgeon gender and stress during the Covid-19 pandemic. Annals of Surgery.

[CR34] Moccia L, Janiri D, Pepe M, Dattoli L, Molinaro M, De Martin V, Di Nicola M (2020). Affective temperament, attachment style, and the psychological impact of the COVID-19 outbreak: An early report on the Italian general population. Brain, Behaviour and Immunity.

[CR35] Mondo, M., Sechi, C., & Cabras, C. (2021). Psychometric evaluation of three versions of the Italian perceived stress scale. *Current Psychology,**40*, 1884–1892. 10.1007/s12144-019-0132-8

[CR36] Okruszek, Ł., Aniszewska-Stańczuk, A., Piejka, A., Wiśniewska, M., & Żurek, K. (2020). Safe but lonely? Loneliness, mental health symptoms and COVID-19. *Frontiers in Psychology*, 11, 579181. 10.31234/osf.io/9njps 10.3389/fpsyg.2020.579181 PMC7747668 33343454

[CR3] Parlangeli, O., Guidi, S., Marchigiani, E., Bracci, M., & Liston, P. M. (2020). Perceptions of work-related stress and ethical misconduct amongst non-tenured researchers in Italy. *Science and Engineering Ethics*, *26*, 159–181. 10.1007/s11948-019-00091-6 10.1007/s11948-019-00091-6 30719620

[CR2] Parlangeli, O., Palmitesta, P., Bracci, M., Caratozzolo, M. C., Liston P. M., & Marchigiani, E. (2017). Stress and perceptions of unethical behaviour in academia. ICERI2017, 10th International Conference of Education Research and Innovation,16–19 nov., Seville, Spain. 10.21125/iceri.2017.0586

[CR37] Pedhazur EJ, Schmelkin LP (1991). Measurement, design, and analysis: An integrated approach.

[CR38] Philipsen B, Tondeur J, Pareja Roblin N, Vanslambrouck S, Zhu C (2019). Improving teacher professional development for online and blended learning: A systematic meta-aggregative review. Education Technology Research and Development.

[CR39] Quattrone, F., Borghini, A., Emdin, M., & Nuti, S. (2020). Protecting higher education institutions from COVID-19: Insights from an Italian experience. *Journal of American College Health,**1–2*,. 10.1080/07448481.2020.1791885 10.1080/07448481.2020.1791885 32701399

[CR40] Redman, B. K., & Caplan, A. L. (2017). Improving research misconduct policies. *EMBO Reports*, 18(12), 511–514. . 10.15252/embr.201744110 PMC5376957 28283533

[CR41] Shanafelt TD, West CP, Sloan JA, Novotny PJ, Poland GA, Menaker R, Rummans TA, Dyrbye LN (2009). Career fit and burnout among academic faculty. Archives of Internal Medicine.

[CR42] Singh, J., Sharma, S. K., & Gupta, P. (2021). Physical learning environment challenges in the digital divide: How to design effective instruction during COVID-19? *Communications of the Association for Information Systems*, 48. 10.17705/1CAIS.04818

[CR43] Steneck NH (2006). Fostering integrity in research: Definitions, current knowledge, and future directions. Science and Engineering Ethics.

[CR44] Sun S, Goldberg SB, Lin D, Qiao S, Operaio D (2021). Psychiatric symptoms, risk, and protective factors among university students in quarantine during the COVID-19 pandemic in China. Global Health.

[CR45] Tennant R, Hiller L, Fishwick R, Platt S, Joseph S, Weich S, Parlinson J, Secker J, Stewart-Brown S (2007). The Warwick-Edinburgh Mental Well-being Scale (WEMWBS): Development and UK validation. Health and Quality of Life Outcomes.

[CR46] Wang, C., Pan, R., Wan, X., Tan, Y., Xu, L., Ho, C., & Ho, R. (2020). Immediate psychological responses and associated factors during the initial stage of the 2019 coronavirus Disease (COVID-19). Epidemic among the general population in China. *International Journal of Environmental Research and Public Health*, 17, 1729. 10.3390/ijerph17051729 10.3390/ijerph17051729 PMC7084952 32155789

[CR47] Warttig SL, Forshaw MJ, South J, White AK (2013). New, normative, English-sample data for the Short Form Perceived Scale (Pss-4). Journal of Health Psychology.

[CR48] Watermeyer, R., Crick, T., Knight, C., & Goodall, J. (2021). COVID-19 and digital disruption in UK universities: Afflictions and affordances of emergency online migration. *Higher Education*, 81, 623–641. 10.1007/s10734-020-00561-y 10.1007/s10734-020-00561-y PMC7269686 32836334

[CR49] Wu D, Wu T, Liu Q, Yang Z (2020). The SARS-CoV-2 outbreak: What we know. International Journal of Infectious Diseases.

