# Supplementary material for: University Teachers During the First Lockdown Due to SARS-CoV-2 in Italy: Stress, Issues and Perceptions of Misconduct
Source: Sci Eng Ethics. 2022 Feb 15;28(1):9. doi: 10.1007/s11948-022-00362-9 (PMC8847283; doi:10.1007/s11948-022-00362-9)
Supplement: Supplementary file 1 — Supplementary file1 (DOCX 21 kb) [file 11948_2022_362_MOESM1_ESM.docx]

**Supplementary Material 1. Questionnaire used in the study**

**SEZIONE 1**

**Informazioni generali**

**1. Genere**

M

F

Non dichiara

**2. Età(anni) ________**

**3.Sei residente in Italia?**

**Sì**

**No**

**4. Posizione lavorativa attuale**

Prima fascia

Seconda fascia

Ricercatore

RTDB

RTDA

Assegnista di ricerca

Docente a contratto

5. **Da quanti anni hai un ruolo in accademia (in qualsiasi ateneo, in qualsiasi ruolo, sommando anche più periodi)?**

_____________________

**6. Anzianità di servizio nell’Ateneo**

0-5

6-15

16-25

26 e oltre

**7. Da quanti anni ricopri la tua attuale posizione lavorativa?**

_____________________

**8. Qual è il tuo attuale regime lavorativo**

Part time

Tempo pieno

**9. Area scientifica di riferimento (Aree CUN riaggregate)**

Scienze matematiche, fisiche, chimiche e della terra [01+02+03+04]

Scienze biologiche [05]

Scienze mediche [06]

Ingegneria [08+09]

Scienze umanistiche [10+11]

Scienze giuridiche [12]

Scienze economiche, statistiche, politiche e sociali [07+13+14]

**SEZIONE 2**

**Come consideri l’ambiente in cui hai vissuto nel periodo di quarantena (metà marzo-fine maggio 2020)?**

1. Poco ampio 1 2 3 4 5 Molto ampio
2. Con poche persone 1 2 3 4 5 Con molte persone
3. Posizionato male rispetto a paese/città 1 2 3 4 5 Posizionato bene rispetto a paese/città
4. Aveva quello di cui avevo bisogno 1 2 3 4 5 Mancava quello di cui avevo bisogno

**L’ambiente in cui hai vissuto durante la quarantena (metà marzo-fine maggio 2020) nel complesso era piacevole?**

1. Per niente d’accordo 1 2 3 4 5 Molto d’accordo

**SEZIONE 3**

**Informazioni sulle dotazioni tecnologiche**

**1. In questo periodo di lavoro a distanza, hai avuto accesso ad un computer (o ad un tablet) per la tua attività lavorativa?**

Sì

No

Altro ______________________________________________

**2. Se sì, era un computer a uso esclusivamente personale?**

Sì

No

Altro**_____________________________________________**

**3. Se sì, il computer aveva delle caratteristiche tecniche adeguate?**

Sì

No

Altro**_____________________________________________**

**4. Hai avuto accesso ad Internet dal luogo di permanenza durante la quarantena (metà marzo-fine maggio 2020)?**

Sì

No

Altro ______________________________________________

**5. Se sì, la connessione era adeguata alle tue necessità di collegamento?**

Assolutamente sì 1 2 3 4 5 assolutamente no

**SEZIONE 4**

**Didattica in periodo di quarantena**

**In relazione al periodo di quarantena (metà marzo-fine maggio 2020)**

**Quali aspetti sono risultati più problematici?**

**Gestire gli esami online**

per niente 1 2 3 4 5 molto

**Le relazioni con i colleghi**

per niente 1 2 3 4 5 molto

**Tenere le lezioni online**

per niente 1 2 3 4 5 molto

**Il contatto con gli studenti**

per niente 1 2 3 4 5 molto

**Svolgere attività di carattere amministrativo**

per niente 1 2 3 4 5 molto

**Ottenere un adeguato supporto tecnico o amministrativo**

per niente 1 2 3 4 5 molto

**Svolgere attività di ricerca**

per niente 1 2 3 4 5 molto

**Produzione di pubblicazioni**

per niente 1 2 3 4 5 molto

**SEZIONE 5**

***Questionario PSS 4***

Ti preghiamo di rispondere alle seguenti domande, selezionando per ognuna la risposta che descrive, secondo te, il tuo stato di benessere psico-fisico, considerando il tuo stato di salute

**in relazione al periodo di quarantena (metà marzo-fine maggio 2020)**

1.Con che frequenza hai avuto la sensazione di non essere in grado di avere controllo sulle cose importanti della tua vita?

mai 0 1 2 3 4 molto spesso

2.Con che frequenza ti sei sentito fiducioso sulla tua capacità di gestire i tuoi problemi personali?

mai 0 1 2 3 4 molto spesso

3.Con che frequenza hai avuto la sensazione che le cose andassero come dicevi tu?

mai 0 1 2 3 4 molto spesso

4.Con che frequenza hai avuto la sensazione che le difficoltà si stessero accumulando fino al punto di non poterle superare?

mai 0 1 2 3 4 molto spesso

***Questionario wellbeing (WEMWBS 12 item)***

1. Mi sono sentita/o ottimista riguardo al futuro

Mai 1 2 3 4 5 Sempre

1. Mi sono sentita/o utile

Mai 1 2 3 4 5 Sempre

1. Mi sono sentita/o rilassato

Mai 1 2 3 4 5 Sempre

1. Mi sono sentita/o interessato ad altre persone

Mai 1 2 3 4 5 Sempre

1. Ho avuto grinta da vendere

Mai 1 2 3 4 5 Sempre

1. Ho affrontato bene i problemi

Mai 1 2 3 4 5 Sempre

1. Ho pensato in modo chiaro

Mai 1 2 3 4 5 Sempre

1. Mi sono sentita/o vicino ad altre persone

Mai 1 2 3 4 5 Sempre

1. Mi sono sentita/o sicuro di me

Mai 1 2 3 4 5 Sempre

1. Sono stata/o in grado di prendere decisioni

Mai 1 2 3 4 5 Sempre

1. Mi sono interessata/o a cose nuove

Mai 1 2 3 4 5 Sempre

1. Mi sono sentita/o di buon umore

Mai 1 2 3 4 5 Sempre

**Valutazione dei conflitti e delle sinergie tra vita e lavoro in caso di lavoro domiciliare**

***Equilibrio vita-lavoro (Fisher et al., 2009) ****

Ti preghiamo di leggere con attenzione le seguenti affermazioni e di indicare per ognuna quanto frequentemente ti sei sentito in questo modo **durante il periodo di quarantena (metà marzo-fine maggio 2020)**, utilizzando la scala sotto riportata.

1. **Dovevo rinunciare ad importanti attività personali a causa della quantità di tempo che dovevo dedicare alla mia attività lavorativa.**

mai 1 2 3 4 5 quasi sempre

1. **Quando finivo di lavorare ero troppo stanca/o per fare le cose che avrei voluto.**

mai 1 2 3 4 5 quasi sempre

1. **Spesso dovevo trascurare le mie necessità private/personali per le richieste della mia attività lavorativa.**

mai 1 2 3 4 5 quasi sempre

1. **La mia vita privata risentiva degli impegni di lavoro.**

mai 1 2 3 4 5 quasi sempre

1. **Il lavoro mi rendeva difficile condurre la vita privata come avrei desiderato.**

mai 1 2 3 4 5 quasi sempre

1. **Il lavoro mi dava l’energia per svolgere attività extra che sono importanti per me.**

mai 1 2 3 4 5 quasi sempre

1. **Grazie all’attività lavorativa, a casa ero di umore migliore.**

mai 1 2 3 4 5 quasi sempre

1. **Le attività lavorative mi aiutavano a gestire/affrontare problemi personali e pratici a casa.**

mai 1 2 3 4 5 quasi sempre

1. **La mia vita privata sottraeva delle energie di cui avevo bisogno per lavorare**mai 1 2 3 4 5 quasi sempre
2. **L’attività lavorativa risentiva a causa di tutto quello che succedeva nella mia vita privata**mai 1 2 3 4 5 quasi sempre
3. **Avrei dedicato più tempo al lavoro se non fosse stato per tutto quello che succedeva nella mia vita privata**
   mai 1 2 3 4 5 quasi sempre
4. **Ero troppo stanca/o per lavorare come si deve a causa delle cose che accadevano nella mia vita privata**

mai 1 2 3 4 5 quasi sempre

1. **Quando lavoravo mi preoccupavo per le cose che dovevo fare per la mia vita privata oltre al lavoro**

mai 1 2 3 4 5 quasi sempre

1. **Mi era difficile lavorare bene a causa delle preoccupazioni relative a cose personali**mai 1 2 3 4 5 quasi sempre
2. **Quando lavoravo ero di umore migliore per via di quello che succedeva nella mia vita privata**Mai 1 2 3 4 5 quasi sempre
3. **La mia vita privata mi dava energia per lavorare**Mai 1 2 3 4 5 quasi sempre
4. **La mia vita privata mi aiutava a rilassarmi e ad essere pronta/o per un altro giorno di lavoro**
   Mai 1 2 3 4 5 quasi sempre

*****

**items from 1 to 5 measures Work Interference with Personal Life (WIPL)**

**items from 6 to 8 measures Work Enhancement of Personal Life (WEPL)**

**items from 9 to 14 measures Personal Life Interference with Work (PLIW)**

**items from 15 to 17 measures Personal Life Enhancement of Work (PLEW)**

**SEZIONE 6**

**Aspetti comportamentali**

In questa sezione ti rivolgeremo alcune domande volte ad indagare/rilevare aspetti comportamentali e di relazione nell’ambiente universitario/accademico. Ti preghiamo di rispondere in riferimento alle tue esperienze nell’arco della tua carriera accademica.

***1. Correttezza dell’operato***

**1.** Quanto frequentemente (nella tua carriera accademica) è capitato che tuoi colleghi **attuassero comportamenti poco corretti nella loro attività**

(come utilizzare pezzi di pubblicazioni di altri, manipolare in senso confermativo i dati di uno studio, aggiungere indebitamente nomi alle pubblicazioni, ecc.)

mai 1 2 3 4 5 sempre

**1.b. Rispetto a quanto accade di solito, nel periodo della quarantena** (metà marzo-fine maggio 2020) **pensi che questo tipo di comportamenti sono diminuiti o aumentati?**

molto diminuiti -2 -1 0 1 2 molto aumentati

***2. Relazioni con i colleghi***

**2.** Quanto frequentemente è accaduto che **tuoi colleghi** adottassero **comportamenti poco corretti nei confronti di altri colleghi?**

(come ad esempio scambiarsi favori, favorire avanzamenti di carriera, non scambiare informazioni rilevanti, parlare in termini poco lusinghieri dialtri, includere/non includere nelle pubblicazioni, escludere dal proprio "gruppo", ecc.)

mai 1 2 3 4 5 sempre

**2.b. Rispetto a quanto accade di solito, nel periodo della quarantena** (metà marzo-fine maggio 2020) **pensi che questo tipo di comportamenti sono diminuiti o aumentati?**

molto diminuiti -2 -1 0 1 2 molto aumentati

***3. Relazioni docenti-studenti***

**3.** Quanto frequentemente è capitato che **i docenti si comportassero in modo poco corretto nei confronti degli studenti?**

(ad esempio non supportare i lavori di tesi in commissione, fare favoritismi ed eccezioni in sede d'esame, non fornire loro adeguate spiegazioni ed assistenza, non essere puntuali nella conduzione delle lezioni, non fare ricevimento studenti, ecc.)

mai 1 2 3 4 5 sempre

**3.b Rispetto a quanto accade di solito, nel periodo della quarantena** (metà marzo-fine maggio 2020) **pensi che questo tipo di comportamenti sono diminuiti o aumentati?**

molto diminuiti -2 -1 0 1 2 molto aumentati

***4. Relazioni studenti-docenti***

**4.** Quanto frequentemente è capitato che **gli studenti attuassero comportamenti poco corretti nei confronti dei docenti?**

(come l'invio continuo di e-mail, l'adottare un atteggiamento poco rispettoso o troppo pressante, ecc)

mai 1 2 3 4 5 sempre

**4.b Rispetto a quanto accade di solito, nel periodo della quarantena** (metà marzo-fine maggio 2020) **pensi che questo tipo di comportamenti sono diminuiti o aumentati?**

molto diminuiti -2 -1 0 1 2 molto aumentati
