# Supplementary material for: University Teachers During the First Lockdown Due to SARS-CoV-2 in Italy: Stress, Issues and Perceptions of Misconduct
Source: Sci Eng Ethics. 2022 Feb 15;28(1):9. doi: 10.1007/s11948-022-00362-9 (PMC8847283; doi:10.1007/s11948-022-00362-9)
Supplement: Supplementary file 2 — Supplementary file2 (DOCX 18 kb) [file 11948_2022_362_MOESM2_ESM.docx]

**Supplementary Material 2. Confirmatory Factor Analysis of the Italian translation of the scale for measuring interference between work and personal life.**

*Confirmatory Factor Analysis Standardized Regression Weights for Italian translation of Work/Nonwork Scale (Fisher et al., 2009). Original English item in italic.*

|  | **WIPL** | **WEPL** | **PLIW** | **PLEW** |
| --- | --- | --- | --- | --- |
| **Dovevo rinunciare ad importanti attività personali a causa della quantità di tempo che dovevo dedicare alla mia attività lavorativa.**  *(I have to miss out on important personal activities due to the amount of time I spend doing work)* | 0.845 |  |  |  |
| **Quando finivo di lavorare ero troppo stanca/o per fare le cose che avrei voluto**  *(I come home from work too tired to do things I would like to do)* | 0.882 |  |  |  |
| **Spesso dovevo trascurare le mie necessità private/personali per le richieste della mia attività lavorativa**  *(I often neglect my personal needs because of the demands of my work)* | 0.935 |  |  |  |
| **La mia vita privata risentiva degli impegni di lavoro**  *(My personal life suffers because of my work)* | 0.935 |  |  |  |
| **Il lavoro mi rendeva difficile condurre la vita privata come avrei desiderato** *(My job makes it difficult to maintain the kind of personal life I would like)* | 0.925 |  |  |  |
| **Il lavoro mi dava l’energia per svolgere attività extra che sono importanti per me**  *(My job gives me energy to pursue activities outside of work that are important to me)* |  | 0.735 |  |  |
| **Grazie all’attività lavorativa, a casa ero di umore migliore**  *(Because of my job, I am in a better mood at home)* |  | 0.896 |  |  |
| **Le attività lavorative mi aiutavano a gestire/affrontare problemi personali e pratici a casa**  *(The things I do at work help me deal with personal and practical issues at home)* |  | 0.830 |  |  |
| **La mia vita privata sottraeva delle energie di cui avevo bisogno per lavorare**  *(My personal life drains me of the energy I need to do my job)* |  |  | 0.838 |  |
| **L’attività lavorativa risentiva a causa di tutto quello che succedeva nella mia vita privata**  *(My work suffers because of everything going on in my personal life)* |  |  | 0.922 |  |
| **Avrei dedicato più tempo al lavoro se non fosse stato per tutto quello che succedeva nella mia vita privata**  *(I would devote more time to work if it weren’t for everything I have going on in my personal life)* |  |  | 0.883 |  |
| **Ero troppo stanca/o per lavorare come si deve a causa delle cose che accadevano nella mia vita privata**  *(I am too tired to be effective at work because of things I have going on in )* |  |  | 0.916 |  |
| **Quando lavoravo mi preoccupavo per le cose che dovevo fare per la mia vita privata oltre al lavoro**  *(When I’m at work, I worry about things I need to do outside work)* |  |  | 0.812 |  |
| **Mi era difficile lavorare bene a causa delle preoccupazioni relative a cose personali**  *(I have difficulty getting my work done because I am preoccupied with personal matters at work)* |  |  | 0.854 |  |
| **Quando lavoravo ero di umore migliore per via di quello che succedeva nella mia vita privata**  *(I am in a better mood at work because of everything I have going for me in my personal life)* |  |  |  | 0.472 |
| **La mia vita privata mi dava energia per lavorare**  *(My personal life gives me the energy to do my job)* |  |  |  | 0.923 |
| **La mia vita privata mi aiutava a rilassarmi e ad essere pronta/o per un altro giorno di lavoro**  *(My personal life helps me relax and feel ready for the next day’s work)* |  |  |  | 0.887 |

*Note. N* = 547. All regression weights are statistically significant. Model fit statistics for the four-factor model were as follows, Chi2(113, *N* = 547) = 626.4, normed, nonnormed, and comparative fit indexes are .99, .99, and .99, respectively; root-mean-square-error of approximation = .091. Standardized Root Mean Square Residual = 0.069. WIPL= work interference with personal life; PLIW = personal life interference with work; WEPL = work enhancement of personal life; PLEW = personal life enhancement of work.
